# Intratumoral gene expression of dihydrofolate reductase and folylpoly-c-glutamate synthetase affects the sensitivity to 5-fluorouracil in non-small cell lung cancer

**DOI:** 10.1007/s12672-021-00413-w

**Published:** 2021-06-25

**Authors:** Kayo Sakon, Masato Sasaki, Kaede Tanaka, Tae Mizunaga, Keita Yano, Yuuko Kawamura, Akitoshi Okada, Takeshi Ikeda, Sawaka Tanabe, Atsushi Takamori, Narihisa Yamada, Kouichi Morioka, Takaaki Koshiji

**Affiliations:** 1 Department of Surgery (II), Faculty of Medical Sciences, University of Fukui, 23-3 Shimoaizuki, Matsuoka Eiheijichou, Fukui, 910-1193 Japan; 2 Department of Thoracic Surgery, National Center for Global Health and Medicine, 1-21-1 Toyama Shinjyuku-ku, Tokyo, 162-8655 Japan

**Keywords:** Histoculture drug response assay, Reverse-transcriptase polymerase chain reaction, Thymidylate synthase, Dihydropyrimidine dehydrogenase, Orotate phosphoribosyltransferase

## Abstract

**Background:**

Various factors related to the sensitivity of non-small cell lung carcinoma (NSCLC) to 5-fluorouracil (5-FU) have been reported, and some of them have been clinically applied. In this single-institutional prospective analysis, the mRNA expression level of five folic acid-associated enzymes was evaluated in surgical specimens of NSCLC. We investigated the correlation between the antitumor effect of 5-FU in NSCLC using an anticancer drug sensitivity test and the gene expression levels of five enzymes.

**Materials and methods:**

Forty patients who underwent surgery for NSCLC were enrolled, and the antitumor effect was measured using an in vitro anticancer drug sensitivity test (histoculture drug response assay) using freshly resected specimens. In the same sample, the mRNA expression levels of five enzymes involved in the sensitivity to 5-FU were measured in the tumor using real-time PCR. The expression levels and the result of the sensitivity test were compared.

**Results:**

No correlation was found between dihydropyrimidine dehydrogenase (*DPD*), orotate phosphoribosyltransferase (*OPRT*), or *DPD/OPRT* expression and the antitumor effects of 5-FU. On the other hand, a correlation was found between thymidylate synthase (*TS*), folylpoly-c-glutamate synthetase (*FPGS*), and dihydrofolate reductase (*DHFR*) expression and 5-FU sensitivity.

**Conclusion:**

Expression of *FPGS* and *DHFR* may be useful for predicting the efficacy of 5-FU-based chemotherapy for NSCLC.

## Introduction

5-fluorouracil (5-FU) is an antimetabolite that is widely used to treat various solid cancers including non-small cell lung cancer (NSCLC) [1, 2] In Japan, an oral 5-FU prodrug has been developed. At present, UFT® and TS-1® are indicated in Japan for NSCLC and are often used as one of the drugs in postoperative adjuvant chemotherapy or systemic chemotherapy [3, 4] Despite various measures to enhance the effects of 5-FU, the treatment is ineffective in many patients. In addition, 5-FU anticancer agents are less effective in patients with advanced or recurrent NSCLC [2] However, if the therapeutic effect of 5-FU anticancer drugs in cancer patients can be predicted before administration, the disadvantages of unnecessary administration can be prevented. Thus, identification of factors that can predict the effect of drugs before drug administration is important.

5-FU is activated only after it is converted to 5-fluorodeoxyuridine monophosphate (FdUMP). FdUMP inhibits DNA synthesis by forming a ternary complex with Thymidylate synthase (TS), which is an essential enzyme for DNA synthesis, together with 5,10-methylenetetrahydrofolate (5,10-CH2-THF). It has been reported that the main pathway for conversion of 5-FU to FdUMP in tumors is the pathway converted by Orotate phosphoribosyltransferase (*OPRT*) in the presence of phosphoribosylpyrophosphate (PRPP) [5] On the other hand, 5-FU is rapidly degraded by dihydropyrimidine dehydrogenase (*DPD*). Therefore, the expression levels of *OPRT* and *DPD* may be involved in the effects of 5-FU. In addition, 5,10-CH2-THF, which forms a ternary complex with FdUMP and TS, is one of the reduced folic acids and is regulated by dihydrofolate reductase (*DHFR*) and folylpoly-c-glutamate synthetase (*FPGS*) [6] Therefore, *DHFR* and *FPGS* may also be involved in the effects of 5-FU.

Several reports of NSCLC cases have described the sensitivity to 5-FU using clinical samples [7–9], but no clear findings have been obtained. In this study, the level of mRNA expression (*TS, DPD, OPRT, DHFR*, and *FPGS*) of factors associated with 5-FU sensitivity was measured with reverse-transcriptase polymerase chain reaction (RT-PCR), and we used the histoculture drug response assay (HDRA) method to assess sensitivity to 5-FU. We then investigated the correlation between the expression level of each factor and the chemosensitivity result.

## Patients and methods

### Patients

Tissue samples were collected at the time of surgery from 40 patients who underwent surgery at the Department of Surgery (II), University of Fukui Hospital for NSCLC with a tumor size > 20 mm between January 2012 and December 2015. The experimental use of the chemosensitivity test was approved by the Institutional Research Committee, and the trial was approved by the Institutional Review Board for University of Fukui Hospital. All patients were informed of the nature of this study, and written informed consent was obtained.

Fresh specimens were sampled from the primary lesion immediately after surgical resection, immersed in Hank’s solution, and used for in vitro chemosensitivity testing with HDRA. Ten-micrometer-thick slices from the paraffin-embedded specimens were later used for quantitative RT-PCR.

### HDRA

The HDRA was used as an in vitro drug sensitivity test as previously reported. [10, 11] Collagen sponge gels (Gel foam®) manufactured from pig skin were purchased from Pfizer Japan Inc. (Tokyo, Japan). Cancerous portions of specimens were minced into pieces of approximately 10 mg, which were then placed on the prepared collagen surface in 24-well microplates. Plates were incubated for 7 days in the presence of drugs dissolved in RPMI 1640 medium containing 20% fetal calf serum in a humidified atmosphere containing 95% air/5% CO_2_ at 37 °C. 5-FU was provided by Tokyo Chemical Industry Co. Ltd. (Tokyo, Japan) and used at 300 μg/ml. After these specimens were histocultured, 100 μl Hank’s balanced salt solution containing 0.1 mg/ml type I collagenase (Sigma) and 100 μl 9.6 mg/ml 3-(4,5-dimetylthiazol-2-yl)-2,5-diphenyl tetrazolium bromide (MTT) solution dissolved in phosphate-buffered saline were added to each culture well and incubated for another 16 h. Following extraction with dimethysulfoxide, absorbance of the solution in each well was read at 540 nm (control 630 nm) using a microplate reader (Spectra Max M5; Molecular Device LLC, San Jose, CA). Absorbance per gram of cultured tumor tissue (OD/W) was calculated from the mean absorbance of tissue from three culture wells. The tumor tissue weight was determined prior to culture.

The inhibition rate was calculated using the following formula:

$${\text{Inhibition rate }}\left( \% \right){\text{ }} = {\text{ }}\left( {{{1 }} - {\text{mean OD/W of treated well/mean OD/W of control well}}} \right){\text{ }} \times 100$$

### Laser-capture microdissection and real-time RT-PCR (the Danenberg tumor profile [DTP] method)

The quantitative assay of the five genes of interest (*TS, DPD, OPRT, DHFR*, and *FPGS*) using paraffin-embedded sections of resected NSCLC specimens was performed according to the DTP method of Response Genetics (New York, NY, USA) [12]. For every specimen, four sets of 10-µm-thick sections and one set of 5-µm-thick sections were prepared from formalin-fixed, paraffin-embedded tissues. The 5-µm-thick sections were stained with hematoxylin and eosin and examined histologically. The 10-µm-thick sections were stained with nuclear fast red (American Master Tech Scientific, Lodi, CA) and used for laser-capture microdissection (PALM Microsystem, Leica, Wetzlar, Germany) from the upper and lower thirds of tumors, separately. The dissected tissue samples were transferred to reaction tubes containing 400 µl RNA lysis buffer, and RNA was isolated. Finally, cDNA was prepared as described by Lord et al. [13, 14].

Quantification of the five genes of interest (*TS, DPD, OPRT, DHFR*, and *FPGS*) and an internal reference gene (β-actin) was performed using a fluorescence-based real-time PCR system (ABI PRISM 7900 Sequence Detection System, Applied Biosystems, Foster City, CA). The final volume of the reaction mixture was 20 µl. Cycling conditions and the primers and probes were described previously by Matsubara et al. [15] Gene expression was analyzed twice to confirm the reproducibility, and values (relative mRNA levels) were expressed as the ratio between the gene of interest and the internal reference gene (β-actin).

### Examination of the necessity of Gimeracil (5-chloro-2, 4-dihydroxypyridine (CDHP))

NSCLC has high DPD activity [12] and requires the use of CDHP, an inhibitor of DPD. In an anticancer sensitivity test, the sensitivity effect of adding CDHP was also examined. Previous experiments were performed to determine the appropriate concentration of CDHP. The experiment was performed by dividing 5-FU alone into four groups at a concentration obtained by adding 200 μg/ml and 300 μg/ml CDHP to 200 μg/ml and 300 μg/ml 5-FU. At that concentration, 5-FU was used in combination with CDHP, and the results were observed.

### Statistical analysis

Statistical analysis was performed on a personal computer with Stat Mate IV (Ver. IV, ATMS, Japan). The correlations between the mRNA expression of genes examined and clinico-pathological parameters were evaluated with the Pearson product moment correlation coefficient. To evaluate the correlation between two variables, linear regression was performed, and the Spearman rank correlation coefficient was calculated. Probability (*P*) values of less than 0.05 were considered statistically significant.

## Results

### Anticancer sensitivity test (HDRA) for 5-FU in our institution

The anticancer drug sensitivity test for 5-FU in NSCLC has been conducted in our department since 2006, and HDRA at the relevant 5-FU concentration of 300 μg/ml was successfully performed for 419 patients (289 males and 130 females, median age 71.3 years, range 39–82 years).

### Examination of the necessity of CDHP

A test was conducted to examine the results of sensitivity to 5-FU by changing the concentration of CDHP in 29 cases of NSCLC. We found no significant difference in sensitivity between the 5-FU only group (suppression rate 62 ± 17%, n = 29) and the group to which CDHP was added (300 mg; 60 ± 18%, n = 29, 200 mg; 60 ± 17%, n = 23). Therefore, this study was performed using only 5-FU. We also examined the toxicity of CDHP by performing a CDHP alone sensitivity test without 5-FU. CDHP alone showed a very low inhibition rate and no antitumor effect (8 ± 10%, n = 29).

### Patient characteristics

Forty patients (29 men (72.5%) and 11 women (27.5%)) who underwent surgery at our hospital for NSCLC were enrolled (Table 1). The median age was 70 years (range, 47–84 years). The postoperative pathological stage was stage IA in 10 patients (25%), stage IB in nine (22.5%), stage IIA in 10 (25%), stage IIB in four (10%), stage IIIA in five (12.5%), and stage IV in two (5%).

**Table 1:** Patients characteristics

Age(medial value)	
Sex	70 (47–84)
Male	29 (72.5%)
Female	11 (27.5%)
Smoking	
Former and current	30 (75%)
Never	10 (25%)
Performance status	
0	28 (70%)
1	9 (22.5%)
2	1 (2.5%)
3	2 (5%)
Pathologocal stage	
IA	10 (25%)
IB	9 (22.5%)
IIA	10 (25%)
IIB	4 (10%)
IIIA	5 (12.5%)
IV	2 (5%)
Operative procedure	
Lobectomy	35 (87.5%)
Segmentectomy	2 (5%)
Partial resection	3 (7.5%)
Pathologocal finding	
Adeno carcinoma	23 (57.5%)
Squamous cell carcinoma	15 (37.5%)
Others	2 (5%)
Genetic status	
L858R:	7
exon19:	3
ALK Translocation:	1
Wild type:	12
Unexamined:	17

**Table 2:** mRNA expression and inhibition and HDRA results

	mRNA (relative gene expression)					HDRA(%)
	*TS*	*DPD*	*OPRT*	*FPGS*	*DHFR*	Inhibition (%)
N	37	37	37	37	37	37
Mean ± SD	3.69 ± 2.89	1.20 ± 0.637	0.706 ± 1.23	0.885 ± 0.477	2.10 ± 2.50	55.4 ± 13.7
Median (range)	2.56 (0.60–11.2)	1.20 (0.25–2.68)	0.371 (0.10–7.57)	0.769 (0.42–1.54)	1.49 (0.50–4.64)	57 (35–66)

**Table 3:**
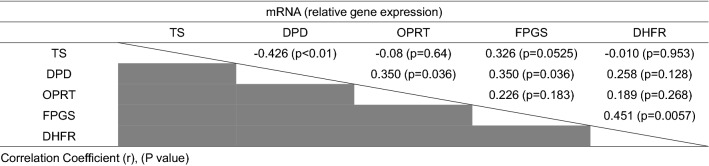
Correlation of TS, DPD, OPRT, FPGS and DHFR

The operative procedure was lobectomy in 35 patients (87.5%), segmental resection in two patients (5%), and partial wedge lung resection in three patients (7.5%). The histology included 23 cases of adenocarcinoma (57.5%), 15 cases of squamous cell carcinoma (37.5%), and two cases (5%) of other types of NSCLC. Regarding EGFR mutation and ALK translocation in adenocarcinoma, seven cases were L858R positive, three cases were exon 19 positive, one case was ALK positive, and 12 cases were wild type (Table 1).

### Comparison of gene expression and the percent inhibition in the anticancer sensitivity test (HDRA) for 5-FU

Gene expression of *TS, OPRT, DPD, FPGS,* and *DHFR* was measured in 39 samples except for one that could not be measured successfully. However, 2 samples did not grow on the HDRA method, so they were excluded. Finally, 37 samples were considered. The mean levels of mRNA of *TS, DPD, OPRT, FPGS,* and *DHFR* in these specimens were 3.69 ± 2.89 (n = 37), 1.200 ± 0.637 (n = 37), 0.706 ± 1.23 (n = 37), 0.885 ± 0.477 (n = 37), and 2.10 ± 2.50 (n = 37), respectively (Table 2). Expression of these genes was not correlated with the age, sex, histopathological type, clinical stage, or driver gene mutation.

Table 3 shows the correlations among *TS, OPRT, DPD, FPGS*, and *DHFR* mRNA for all samples. The mRNA expression levels of *TS* were moderately correlated with those of *DPD* (Correlation Coefficient (r): -0.426, p < 0.01), and those of *DPD* were weakly correlated with those of *OPRT* (r: 0.350, p < 0.036) and *FPGS* (r: 0.350, p < 0.036). The mRNA expression levels of *FPGS* were moderately correlated with those of *DHFR* (r: 0.451, p = 0.0057).

**Fig. 1:**
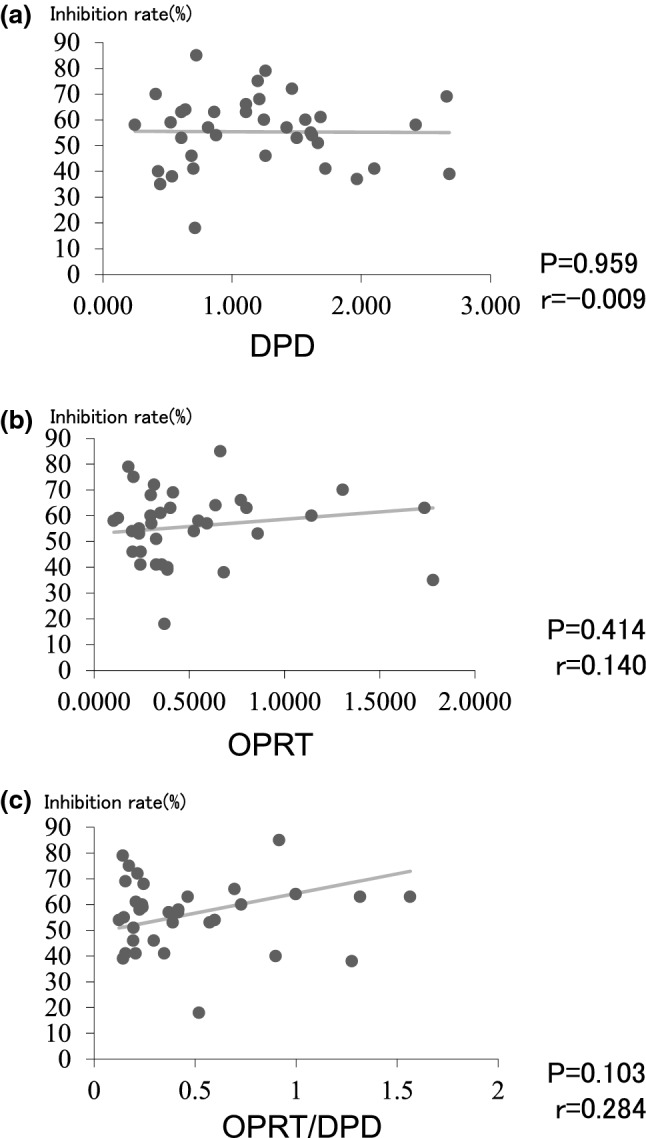
No significant correlation was found between expression of DPD (**a**), OPRT (**b**), and DPD/OPRT (**c**) and the percent inhibition with 5-FU using the HDRA method

**Fig. 2:**
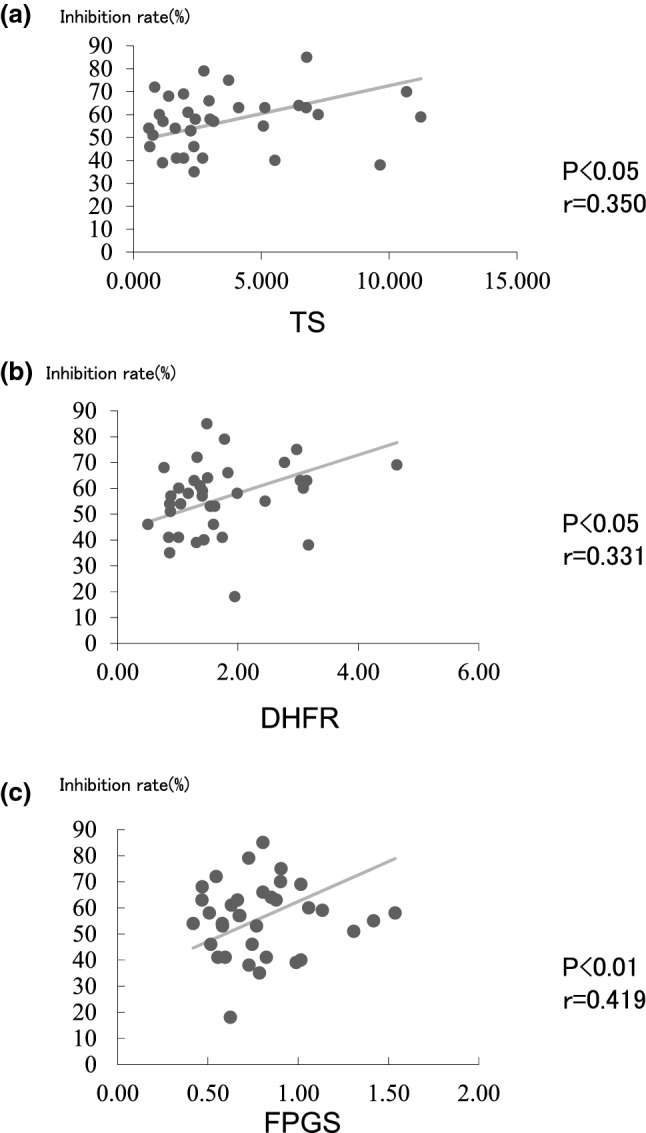
A significant correlation was found between expression of TS (**a**), DHFR (**b**), and FPGS (**c**) and the percent inhibition with 5-FU using the HDRA method

We found no significant correlation between expression of *DPD, OPRT,* or *DPD/OPRT* and the percent inhibition with 5-FU using the HDRA method (Fig. 1). We found a significant correlation between expression of *TS, FPGS,* and *DHFR* and the percent inhibition with 5-FU using the HDRA method (p < 0.05, r = 0.350, p < 0.01, r = 0.418, and p < 0.05, r = 0.331, respectively) (Fig. 2).

## Discussion

To the best of our knowledge, this is the first study to show a correlation between *FPGS* and *DHFR* expression and 5-FU sensitivity results in NSCLC. *FPGS* showed a stronger correlation with 5-FU sensitivity than *DHFR*.

FPGS converts intracellular folic acid and folic acid antagonists, such as methotrexate, into polyglutamic acid, which is retained intracellularly for a long time. Polyglutamylation of intracellular 5,10-methylenetetrahydrofolate enables more efficient formation and stabilization of inhibitory ternary complexes involving TS and metabolites of 5-FU, and it may also increase the cytotoxicity of 5-FU. [6] Two reports have shown a correlation between 5-FU sensitivity and FPGS in colon cancer [6] and breast cancer. [16].

DHFR is the target enzyme of methotrexate, which enters the cell, tightly binds to DHFR, and inhibits the reduction of dihydrofolate to tetrahydrofolate. [17] Only one report has evaluated the activity of DHFR and the antitumor effect of 5-FU. [18] They reported that DHFR mRNA had a high value using a 5-FU-resistant mouse cell line.

5-FU is activated only after it is converted to 5-fluorodeoxyuridine monophosphate (FdUMP). TS, an enzyme that is essential for DNA synthesis, methylates deoxyuridine monophosphate (dUMP) and converts it to deoxythymidine monophosphate (dTMP). Therefore, FdUMP is covalently bonded to TS together with 5,10-methylenetetrahydrofolate (5,10-CH2-THF), which is reduced to folic acid, to form a strong ternary complex, which inhibits DNA synthesis. [19] FPGS acts on the pathway that converts 5,10-CH2-THF from monoglutamate to polyglutamates, the increase in which is indispensable for creating a ternary complex that may affect the sensitizing effect of 5-FU. The function of TS activity is inhibition, and the pool of dTTP, which is a precursor of DNA, is depleted, leading to inhibition of DNA synthesis and cell death. [19].

In this study, the positive correlation between *TS* expression and 5-FU sensitivity is the opposite of the results of other reports of NSCLC. [7, 20–22] Regarding the sensitivity of TS in gastric cancer and colorectal cancer, sporadic reports have shown no correlation or reverse correlation between the sensitivity to 5-FU and increased TS activity. [23, 24] In NSCLC, a positive correlation with TS was reported, but TS activity tends to be lower than in other carcinomas, [12] suggesting that even if TS activity is high, it falls within the range where the effect of 5-FU can be observed.

Regarding the sensitivity of 5-FU, the activities of OPRT and DPD have been well evaluated, and several reports show that they are involved in the sensitivity of NSCLC. [5, 25, 26] The results of this study did not show a correlation between OPRT and DPD expression and 5-FU sensitivity. Although the OPRT/DPD ratio has been reported to be an important predictor of the efficacy of fluoropyrimidine-based chemotherapy for metastatic colorectal cancer, [5] this report on DPD/OPRT showed no significant difference with the sensitivity of 5-FU.

Several reports have described the relationship between *TS, DPD,* and *OPRT* and 5-FU sensitivity in NSCLC. Eguchi et al. [20] evaluated the relationship between response to treatment and immunohistochemical expression levels in patients with advanced NSCLC. Low expression levels of DPD and TS were seen in patients not treated with S-1-carboplatin, which is associated with better response and longer survival in patients treated with paclitaxel-carboplatin. Tumor expression levels of *TS* and *DPD* predict the response to S-1-carboplatin chemotherapy in patients with advanced NSCLC. Nakano et al. [21] reported an immunohistochemical study on the clinical importance of TS, OPRT, and DPD expression using 151 NSCLC specimens resected from patients treated postoperatively with tegafur/uracil (UFT). Patients who had tumors with low TS expression (p = 0.0133), high OPRT expression (p = 0.0145), or low DPD expression (p = 0.0004) had significantly high of 5-year survival rates. Shintani et al. [7] investigated patients using RT-PCR for intratumoral expression and examined the correlation between gene expression and the efficacy of 5-FU in NSCLC. Patients receiving postoperative 5-FU alone (n = 30) comprised the 5-FU group, and those who had only surgery were included in the control group (n = 86). When dichotomized by mean *TS* and *DPD* mRNA levels, patients with low *DPD* tumors receiving 5-FU had significantly better prognosis than those who did not receive adjuvant treatment (p = 0.041). Based on these results, quantification of *TS* and *DPD* mRNA levels can predict the efficacy of 5-FU after surgery in patients with NSCLC.

5-FU is rapidly degraded by DPD. Due to the higher DPD activity in NSCLC compared to other carcinomas, [12] 5-FU alone is less effective, necessitating co-administration of CDHP, for which S-1 was developed. In view of the mechanism of action of 5-FU, the effects of 5-FU are expected to be reduced if expression of the target enzyme TS and the degrading enzyme DPD in tumor tissue is high. CDHP (Gimeracil), which is used in S-1, [27] inhibits DPD. In this study, we found no difference in the sensitivity results even if CDHP was added to 5-FU in the preliminary sensitivity test. This suggests that DPD may not affect the antitumor effect of 5-FU in vitro.

There are several reports that The HDRA method correlates well with the susceptibility of NSCLC to anticancer drugs and its clinical efficacy. [28–30] Further, the usefulness of HDRA has been documented for several other cancer types including gastric cancer [31] and colorectal cancer. [32] This histo-culture method has the advantage of culturing cancer cells while maintaining cell–cell contacts which has good cell viability, and the disadvantage of requiring a certain amount of tissue sample. In the present study, sufficient amount of sample could be obtained from the surgical specimens. Moreover, the high evaluability rate (n = 419, 96.5%) from previous tests for NSCLC conducted at our institution demonstrate that this method is a good alternative for testing the sensitivity of 5-FU in NSCLC.

The limitations of this study are the small number of cases, the single-institution design, and the in vitro results of the anticancer drug sensitivity test. This study pointed out the correaltionship between in vitro sensitivity of NSCLC samples to 5-FU and mRNA level of *FPGS* and *DHFR*, whether or not this can reflect in vivo drug effect needs more investigation. Furthermore, studying the relationship between anticancer effects in NSCLC patients who actually received 5-FU and the expression levels of various factors in the tumors is necessary in clinical study. Another limitation is the in vitro use of specimens obtained during surgery. For unresectable advanced NSCLC, small specimens such as those obtained from bronchoscopy should be used. The feasibility of such transbronchial lung biopsy samples is being investigated. Nakajima et al. [33] used a metastatic lymph node sample obtained with endo-bronchial ultrasound-guided transbronchial needle aspiration in patients with non-small cells to obtain *TS, DPD, TP*, and *OPRT* mRNA. The feasibility of expression analysis should be evaluated. Clinical application is also expected.

Few reports have examined the sensitivity of 5-FU in NSCLC. Our study provides results that will be useful for assessing the sensitivity of 5-FU in future clinical applications.

## Conclusion

The mRNA levels of five folic acid-associated enzymes involved in the antitumor effect of 5-FU were examined in resected NSCLC tumor specimens with RT-PCR, and the in vitro anticancer sensitivity test was performed. In conclusion, *FPGS* and *DHFR* may be involved in 5-FU sensitivity.

Previous studies have reported that *TS, OPRT,* and *DPD* are associated with 5-FU sensitivity. Combined with this result, the folic acid metabolism pathway is also important, and it will be the basis for the development of new anticancer agents using both pathways.

## Data Availability

The dataset is available upon request.

## Abbreviations

HDRA: Histoculture Drug Response Assay,
DPD: Dihydropyrimidine dehydrogenase
OPRT: Orotate phosphoribosyltransferase
TS: Thymidylate synthase
CDHP: 5-Chloro-2,4-dihydroxypyridine
RT-PCR: Reverse-transcriptase polymerase chain reaction
NSCLC: Non-small cell lung carcinoma
5-FU: 5-Fluorouracil
FPGS: Folylpoly-c-glutamate synthetase
DHFR: Dihydrofolate reductase
FdUMP: 5-Fluorodeoxyuridine monophosphate
dUMP: Deoxyuridine monophosphate
dTMP: Deoxythymidine monophosphate
dTTP: Deoxythymidine triphosphate
5,10-CH2-THF: 5,10-Methylenetetrahydrofolate
